# Effects of Soaking and Germination Treatments on the Nutritional, Anti-Nutritional, and Bioactive Characteristics of Adzuki Beans (*Vigna angularis* L.) and Lima Beans (*Phaseolus lunatus* L.)

**DOI:** 10.3390/foods13091422

**Published:** 2024-05-06

**Authors:** Qurat Ul Eain Hyder Rizvi, Raquel P. F. Guiné, Naseer Ahmed, Mohd Aaqib Sheikh, Paras Sharma, Imran Sheikh, Ajar Nath Yadav, Krishan Kumar

**Affiliations:** 1 Department of Food Technology, Dr. Khem Singh Gill Akal College of Agriculture, Eternal University, Baru Sahib, Sirmour 173101, India; syeda15994@gmail.com (Q.U.E.H.R.); naseerfst@gmail.com (N.A.); sheikhaaqib111@gmail.com (M.A.S.); 2 CERNAS Research Centre, Polytechnic University of Viseu, 3504-510 Viseu, Portugal; 3 Department of Food Process Engineering, National Institute of Technology, Rourkela 769005, India; 4 Department of Food Technology, Mizoram University, Aizawl 796004, India; parassharma19791@gmail.com; 5 Department of Biotechnology, Dr. Khem Singh Gill Akal College of Agriculture, Eternal University, Baru Sahib, Sirmour 173101, India; imran@eternaluniversity.edu.in (I.S.); ajar@eternaluniversity.edu.in (A.N.Y.); 6 Department of Food Technology, Rajiv Gandhi University, Doimukh 791112, India

**Keywords:** antioxidant activity, anti-nutrients, bioactive components, germination, nutritional compounds, soaking

## Abstract

Lima beans (*Phaseolus lunatus*) and adzuki beans (*Vigna angularis*) are some of the most nutritious underutilized pulses that are significant in being used as basic ingredients for the preparation of various food products. The present study aimed to determine the impact of soaking and germination on nutritional and bioactive components, in vitro protein digestibility, reducing power, metal chelating capacity, antioxidant activity, and anti-nutritional components of lima and adzuki beans. The findings showed that during the germination treatment, the in vitro protein digestibility of lima and adzuki beans increased by 14.75 and 10.98%, respectively. There was an increase in the antioxidant activity of lima beans by 33.48% and adzuki beans by 71.14% after 72 h of germination, respectively. The reducing power assay of lima and adzuki beans indicated an increase of 49.52 and 36.42%, respectively, during germination. Similarly, the flavonoid and metal chelating activity increased in lima and adzuki beans after 72 h of germination. In contrast, the anti-nutrients, such as phytic acid, tannin content, and trypsin inhibitor activity, decreased significantly *p* < 0.05 after 72 h of germination. These results are encouraging and allow for utilizing the flour obtained from the germinated beans in functional bakery products, which can contribute to eradicating protein deficiency among some population groups. At the same time, promoting soaking and germination of the beans as a way to enhance the nutritional quality and reduce anti-nutrients can contribute to the interest in these underutilized pulses. They could be seen as an additional tool to improve food security.

## 1. Introduction

The most serious problem the developing countries are facing is protein-energy malnutrition. The developing world is facing a major challenge in providing nutritious, safe, and wholesome food for undernourished and poor populations. The search for reliable and affordable sources of protein derived from plants has been prompted by the unpredictability of supply, high costs, and scarcity of foods rich in protein from animals in underdeveloped and developing nations. Some of the underutilized and wild legumes are found to contain rich nutraceutical value. Furthermore, the presence of various anti-nutritional components restricts their utilization in food products, and these can be eliminated by employing certain processing methods like soaking and germination [1].

Among legumes, lima beans (*Phaseolus lunatus*) and adzuki beans (*Vigna angularis*) are considered underutilized pulses in India. Lima beans and adzuki beans are herbaceous plants belonging to the family Fabaceae. Lima beans are also referred to as faba beans, sugar beans, and butter beans. Lima beans are a great source of carbohydrates, dietary fibers, and proteins but contain lesser amounts of fat. It is rich in riboflavin, vitamin B6, thiamin, and niacin, which act as coenzymes for the oxidation of carbohydrates, proteins, and fats [2]. Lima beans can be utilized as whole beans and can also be converted into flour, which can be incorporated into conventional flour for better nutrient content. Lima beans are still underutilized pulses despite having a great nutritional profile. Matured lima bean seeds possess various benefits to human health. Despite various nutritional benefits, both lima and adzuki beans contain various anti-nutrients like phytic acid, tannins, trypsin inhibitors, oxalate, haematoglutinins, and cyanides, which interfere with the utilization and absorption of numerous micronutrients, thereby decreasing the nutritive value and protein digestibility of foods [3].

Adzuki beans are a traditional crop that plays a great role in the sustainability of agriculture and food protein supply. It is known for its traditional therapeutic uses. Adzuki beans have raised global interest not only because of their nutritional properties, health benefits, and processing qualities but also due to their gluten-free nature. Various studies reveal the highest nutrient profile of adzuki and have considered it to be a functional food for disease prevention and health promotion [4]. Adzuki beans are also known for their therapeutic benefits as they are anti-inflammatory, anti-diabetic, and antioxidant. They are also used as herbal medicine that controls weight, maintains health, and acts as a functional food material [5]. Eliminating or reducing these anti-nutrients to a safe level enhances the nutritive value of the food and also ensures the effective use of these legumes for human consumption. 

Conventionally, grains are hydrated in water by soaking [6], which has been shown to be effective in both reducing and eliminating the anti-nutrients found in cereals and pulses [7]. Trypsin inhibitors, phytic acid, and other anti-nutrients that are either fully or partially soluble in water can be reduced in concentration by soaking cereals and legumes for 12 to 18 h, according to multiple studies [6,8]. Rizvi et al. [9] reported that the soaking and germination techniques were found to decrease the anti-nutritional components such as phytic acid and tannin contents in processed pigeon pea grains. There was an increase in the antioxidant activity and the total phenolic contents of germinated pigeon pea grains.

Various investigations have recommended that anti-nutritional factors in beans or legumes can be eliminated or decreased by many processing techniques like germination, fermentation, boiling, cooking, and soaking. Germination is a commonly used conventional technique that increases some anti-nutritional components and improves bioactive components while improving nutrient digestibility [9]. During the germination and soaking process, certain alterations could occur within the seed, and these vary depending on the variety of the seed and the germination condition. Germination is known to be the best technique for reducing the anti-nutritional compounds of pulses [10]. 

Chauhan et al. [11] studied the effect of soaking, germination, fermentation, and roasting on the nutrients, anti-nutrients, and bioactive components of black soybean. The results revealed that the phenolic contents augmented significantly (*p* < 0.05) in germination, fermentation, and roasting. The antioxidant activity of processed grains increased significantly (*p* < 0.05) during germination and fermentation. The anti-nutritional compounds, such as phytic acid and tannin contents, decreased significantly during processing treatments. The consumption of sprouts has increased because of convenience and complex physiological changes that increase the nutritional profile and reduce anti-nutrients that interfere with absorption. 

In order to improve the nutritional quality of functional food products made from these underutilized pulses, the current research was designed to examine the impact of soaking and germination processing treatments on the nutritional, anti-nutritional, and bioactive potential of these pulses. This was performed in light of the therapeutic and nutritional benefits of these underutilized pulses as well as the significance of these processing treatments. Because processed grains have fewer anti-nutritional components, this study also supports the use of traditional processing methods to increase the nutritional value and bioavailability of micronutrients from underutilized pulses.

## 2. Materials and Methods

### 2.1. Materials 

The lima beans (American white variety) and adzuki beans (HPU-51) used in the present study were procured from Bombay super seeds, Dist Rajkot (Gujrat), India, and CSK Palampur, Himachal Pradesh, respectively. In the current investigation, ultrapure-grade chemicals and reagents were employed. These were acquired from typical chemical suppliers, including Sigma-Aldrich, Bangalore, India; Hi-Media, Thane, India; Qualigens, Mumbai, india and Merck, Mumbai India. The 3 kg seeds of each of the lima and adzuki beans were procured, and 250 g seeds of each bean type were taken for processing treatments at different time intervals.

### 2.2. Methods

#### 2.2.1. Soaking and Germination Process

The soaking and germination of lima and adzuki beans were carried out using the procedures outlined by Egli et al. [12]. After eliminating any foreign particles, distilled water was used to soak the grains in a 1:5 ratio. This process was performed for both lima and adzuki beans. The seeds were soaked at room temperature for 12 (S12) and 24 (S24) h, after which they were dried for 24 h at 40 °C in a hot air oven. After being placed in airtight pouches for additional analysis, the grains were kept cold at 4 °C. 

**Figure 1:**
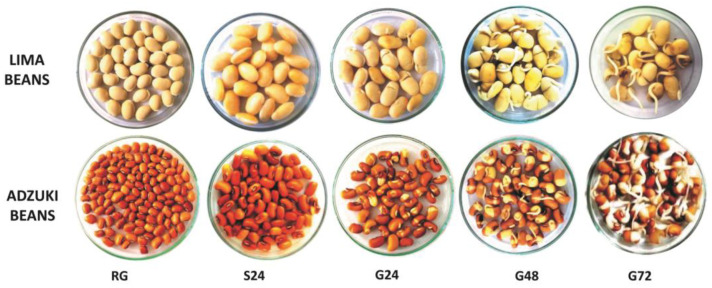
Soaking and germination treatments of lima and adzuki beans at different time intervals (RG—raw grains; S 24—soaking for 24 h; G24—germination for 24 h; G48—germination for 48 h; G72—germination for 72 h).

After steeping, the seeds were drained and covered with the damp muslin cloth, placed in a seed germinator (Western Agro, Helix Technology), and removed at intervals of 24, 48, and 72 h. The germination was carried out for 0 (control), 24 (G24), 48 (G48), and 72 h (G72) in the absence of light at a temperature of 25 °C and 80% relative humidity. Following each germination time treatment, the seeds were dried in a hot air oven at 40 °C for 24 h and ground into fine flour with a laboratory flour mill (SANCO). After that, the flour made from germinated pulses was kept at 4 °C until further examination (Figure 1). The seeds of both the lima and adzuki beans at 0 h without any treatment were kept as a control for comparison with samples treated with soaking and germination treatment at different time intervals.

#### 2.2.2. Physical and Functional Characteristics

The evaluation of the physical characteristics of raw, soaked, and germinated adzuki and lima beans was carried out using standard procedures. Physical parameters like the thousand-grain weight (TGW) were determined by measuring the weight of a thousand grains of adzuki and lima beans and expressed in grams [13]. The length, breadth, and thickness were determined with the help of the Vernier caliper. Bulk density (BD) was evaluated according to the method of Huang et al. [14], and tap density (TD) was evaluated as per the method given by Jones et al. [15]. The water absorption capacity (WAC) was determined as per the methodology described by Sosulski [16], and the oil absorption capacity (OAC) was determined by Kaur et al. [17] after converting grains to flour. The water solubility index (WSI) was estimated using the method of Stojceska et al. [18]. The color characteristics, such as L*, a*, and b* values, were estimated with the help of a chromameter (CR-400, Konika Minolta, Tokyo, Japan).

#### 2.2.3. Chemical Characteristics

The hot air oven method, as described by AOAC [19], was used to estimate the moisture content (%) of the grains. The crude fiber was estimated using Fibroplus FBS 08P (Pelican Inc., Torrance, CA, USA), crude fat was estimated using Soxoplus SPS 06 AS (Pelican Inc.), and crude proteins were estimated using Kjelodist CAS VA (Pelican Inc.). The ash content was calculated using the procedures outlined by Ranganna [20]. The analysis of mineral components was conducted using an Atomic Absorption Spectrometer (AA240FS, Agilent Technology, CA, USA) to determine the presence of iron, zinc, manganese, and copper [19]. By subtracting the measured amounts of moisture, crude protein, ash, crude fat, and crude fiber from 100, the total carbohydrate contents were determined. The factors of 4.0, 9.1, and 4.2 kcal/g for crude protein (Nx6.25), fats, and carbohydrates, respectively, were used to estimate the calorific value (Kcal/100 g) [21]. The methodology, as defined by Shastry and John [22], was used to assess the in vitro protein digestibility. 

Flavonoid content was determined using the method described by Lahlou et al. [23]. The 2.5 g sample was added to a 10 mL distilled water solution and kept overnight at room temperature (37 °C). The mixture was centrifuged at 8000 rpm for 15 min, and the clear supernatant was obtained. One mL of 2% AlCl_3_ was dissolved in 80% methanol, mixed with the 1 mL of extract solution, and incubated for 1 h at 37 °C. Absorbance was measured at 415 nm using a spectrophotometer. Total flavonoid contents were determined using a standard curve of rutin. The results were expressed as mg/100 g rutin equivalent (RE) of the sample. The antioxidant activity (%) was calculated using the method described by Bouaziz et al. [24] by measuring the 2,2-diphenyl-1-picrylhydrazyl (DPPH) radical scavenging capacity. 

The metal chelating activity and reducing capacity were calculated using the procedure as defined by Sharma and Gujral [25]. In the case of metal chelating activity, 0.4 mL FeSO_4_ and 0.8 mL ferrozine were subsequently added into 0.4 mL of plant extract. The mixture was incubated at room temperature for 10 min, and the absorbance of the mixture was measured at 562 nm. The metal chelating activity was calculated using the following formula:

$$Metal\;chelating\;activity\left(\%\right)=\frac{Absorbance\;of\;control-absorbance\;of\;sample}{Absorbance\;of\;control}\times 100$$

For estimation of reducing capacity, the 2.5 mL of phosphate buffer, 2.5 mL of potassium ferricyanide, and 2.5 mL of TCA were added to 1 mL of sample extract and incubated at 50 °C for 20 min. The above mixture was centrifuged at 3000 rpm for 10 min. An amount of 0.5 mL of ferric chloride was added to the above mixture, and the absorbance was measured at 700 nm using a spectrophotometer. A blank was prepared without adding extract. Ascorbic acid at various concentrations (10–100 μg/mL) was used as standard. The increased absorbance of the reaction mixture indicates an increase in reducing capacity (%).

The tannin contents were estimated using the method of Saxena et al. [26], while the trypsin inhibitor activity (TI) was ascertained using the method of Kakade et al. [27]. The phytic acid was assessed using the methodology of Gao et al. [28] with minor modifications. 

The total phenolic content was determined using a Folin–Ciocalteu reagent, followed by a method of Ainsworth and Gillespie [29] with minor modifications. A volume of 0.5 mL of the plant extract was mixed with 2 mL of the Folin–Ciocalteu reagent (diluted 1:10 with deionized water) and neutralized with 4 mL of sodium carbonate solution (7.5%, *w*/*v*). Gallic acid was used as a reference standard for plotting the calibration curve. The color developed by the incubation of the reaction mixture for 30 min was measured at an absorbance of 765 nm using a visible spectrophotometer (711-S NV). The total phenolic contents were assessed from the linear equation of a standard curve prepared with Gallic acid. The content of total phenolic compounds expressed as mg/100 g gallic acid equivalent (GAE) of dry extract.

#### 2.2.4. FT-IR (Fourier Transform Infrared Spectroscopy) Analysis

The FTIR spectroscopic analysis was carried out using a Fourier-transform spectrophotometer (PerkinElmer, Waltham, MA, USA) in the range of 600–4000 cm^−1^.

#### 2.2.5. Statistical Analysis

All experiments were performed in triplicate, and the data collected during the study were subjected to one-way analysis of variance (ANOVA) using the IBM SPSS Statistics 26 software. Based on Duncan’s Multiple Range test post hoc analysis, differences were deemed significant at the *p* < 0.05 level, and values in the tables were expressed as mean ± standard deviation. Principal component analysis (PCA) of all analytical variables was performed using Minitab 16 statistical software.

## 3. Results and Discussion

### 3.1. Physical Properties

The moisture content of lima and adzuki beans was 7.29 and 12.32%, respectively. Farinde et al. [30] reported 7.50% moisture content in lima beans. The initial higher moisture content of grains during storage decreases the germination capacity of grains [31]. The length, width, thickness, and thousand-grain weight (TGW) observed in lima beans were 13.26 mm, 9.87 mm, 9.16 mm, and 699.65 g, respectively. Purwanti and Fauzi [32] reported 15.7 and 10.8 mm of length and width in lima beans, and the TGW observed was 775 g. Similarly, the length, width, thickness, and TGW observed in adzuki beans were 4.32 mm, 2.73 mm, 2.36 mm, and 61.27 g, respectively. Agarwal and Chauhan [4] reported 4.2 mm and 3 mm of length and width, respectively, in adzuki beans. Wu et al. [33] found 58.27 g of TGW in adzuki beans, whereas Yadav et al. [26] reported 74.87 g of TGW in adzuki beans (Table 1).

### 3.2. Functional Properties

The bulk density and tap density observed in lima beans were 0.93 and 1.26 g/cm^3^, and in adzuki beans, the values were observed as 0.86 and 1.16 I g/cm^3^, respectively (Table 1). Yadav et al. [34] observed 0.76 g/mL of bulk density in adzuki beans. The water absorption capacity (WAC) and oil absorption capacity (OAC) were observed as 2.21 and 1.66 mL/g, respectively, in lima beans and 2.89 and 1.81 mL/g, respectively, in adzuki beans. The study carried out by Siddiq et al. [35] reported 2.25 and 1.52 g/g of WAC and OAC, respectively, in red kidney beans. The water solubility index (WSI) of lima and adzuki beans was observed to be 20.23 and 23.23%. Yellavila et al. [3] reported 21.01% WSI in lima beans. The swelling capacities observed in lima and adzuki beans were 82.70 and 70.06%, respectively. The color characteristics such as L*, a*, and b* values observed in lima beans were 82.34, −0.56, 8.07, and that in adzuki beans were 44.14, 4.33, and 0.86, respectively. Woo et al. [36] observed 68.97% of swelling capacity in adzuki beans and reported 43.66, 5.07, and 0.89 L*, a*, and b* values in adzuki beans (Table 1).

### 3.3. Principal Component Analysis

**Figure 2:**
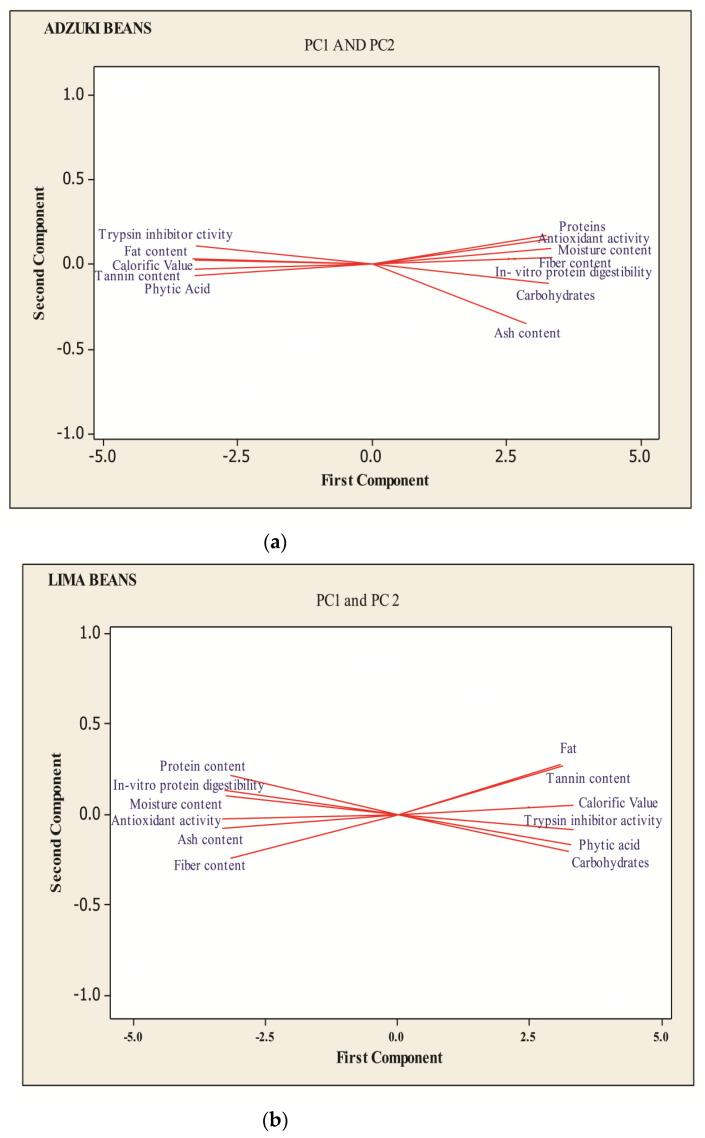
Projections of the variables on the factor plane for adzuki (**a**) and lima beans (**b**).

The results of principal component analysis (PCA) of all analytical variables are presented in Figure 2 and Table 2.

Using the Kaiser criterion, the principal components (PCs), with eigenvalues greater than one (11.245 in lima beans and 11.344 and 4.66 in adzuki beans), were selected. The principal components from 1 to 4 contributed to more than 100 percent of the difference in the selected samples. The PC1, PC2, PC3, and PC4 reported 93.70, 98.80, 100, and 100% for lima beans and 94.50, 98.40, 100, and 100 percent of the variance for adzuki beans, respectively. Out of the four main components reported, the PC1 reported 93.70% of the variance for lima beans and 94.50% of the variance for adzuki beans, which was mostly dominated by calorific value in the case of lima beans and crude fiber in the case of adzuki beans shown in bold in Table 2. The second component was dominated by fat in the case of lima beans and protein in the case of adzuki beans, while protein content and tannin dominated the third component for lima beans and adzuki beans, respectively, and the last component was dominated by calorific value and fat content in case of lima and adzuki beans, respectively.

Therefore, in the current results, most of the proximate components were important variables that had the ability to discriminate with other physico-chemical properties. Figure 2 shows that all samples on the upper side of PC1 were related to the calorific value, while other samples placed on its upper left were related to proximate and tannins. Thus, the proximate composition could be used to differentiate the samples from each other.

### 3.4. Chemical Characteristics of Lima and Adzuki Beans

The fat, protein, ash, and fiber content observed were 1.47, 18.59, 3.78, and 6.55%, respectively, in lima beans and 3.16, 19.56, 3.29, and 5.79%, respectively, in adzuki beans (Table 3 and Table 4). Farinde et al. [30] reported 7.50, 1.22, 22.24, 4.68, and 6.85% of moisture, fat, protein, ash, and fiber contents, respectively, in lima beans, and Yu-Wei and Wang [37] reported 3.1, 22.65, 1.87, and 3.41% of ash, protein, fat, and fiber content in raw adzuki beans.

The in vitro protein digestibility observed in lima and adzuki beans was 67.85 and 70.56%. The results observed by Alonso et al. [38] showed 68.1% in vitro protein digestibility in kidney beans. The phenolic and flavonoid content observed in raw lima beans was 110.43 and 20.16 mg/100 g, and the same in adzuki beans were observed as 22.78 and 43.38 mg QE/100 g, respectively. Khang et al. [39] reported 12.21 mg/100 g of phenolic content in raw adzuki beans. Meanwhile, Sombié et al. [40] reported 23.95 mg QE/100 g of flavonoid content in raw cowpeas seeds. The tannin content found in raw lima and adzuki beans was 10.70 and 6.36 mg/100 g, respectively. Similar results were found by Farinde et al. [30], who reported 9.80 mg/100 g of tannin content in raw lima beans. Phytic acid content found in lima and adzuki beans was 20.48 and 7.12 mg/100 g. Similar results were found by Adeparusi [41], who reported 19.0 mg/100 g of phytic acid in raw lima beans.

The antioxidant activity and reducing activity found in raw lima and adzuki beans were found to be 35.48, 30.55, and 9.74, 32.77%, respectively. Similar results were found by Yu-Wei and Wang [37], who reported 36.24 and 10.82% of antioxidant activity in faba beans and adzuki beans, respectively. Similarly, Granito et al. [42] found 49.78% of antioxidant activity in lima beans. The copper, iron, zinc, and manganese content found in raw lima beans were 4.17, 4.04, 2.45, and 2.36 ppm, and that in adzuki beans were 1.22, 5.16, 4.49, and 1.57 ppm, respectively. Similar results were observed by Jayalaxmi et al. [43], who observed 4.30, 5.40, 2.64, and 2.63 mg/100 g of copper, iron, zinc, and manganese content in raw lima beans.

### 3.5. Effect of Processing Treatments on Nutritional, Anti-Nutritional, and Bioactive Characteristics

#### 3.5.1. Nutritional Characteristics and In Vitro Protein Digestibility

The moisture content increased in lima beans from 7.29 (RG) to 7.74% (G72) and 12.32 (RG) to 14.57% (G72) in adzuki beans, respectively (Table 3 and Table 4). The moisture content increased by 1.23 and 6.17% in lima beans after soaking and germination treatments, respectively. Similarly, it increased by 9.00 and 18.26% after soaking and germination treatments, respectively, in adzuki beans. Farinde et al. [36] reported a 4.66% rise in moisture content in germinated lima beans, whereas Mubarak [44] reported a 13.84% increase in moisture content in mung bean sprouts. The increase in the moisture content may be attributed to the absorption of water from the surroundings during soaking and germination of beans to commence the metabolic processes [45].

The protein content in lima beans increased from 18.59 (RG) to 21.92% (G72 h) and from 19.56 (RG) to 23.46% (G72 h) in adzuki beans. The protein content in lima and adzuki beans increased by 3.01%, 17.19%, and 8.69%, 19.93%, respectively, after soaking and germination treatments. Jayalaxmi et al. [43] observed an increase of 4.47 and 7.72% in protein content, respectively, after soaking and germination treatments in lima beans. The soaking and germination increased the protein content in the lima beans due to the induction of hydrolytic enzymes, which increased the metabolic activity of protein synthesis in seeds. The reawakening of protein synthesis upon imbibition leads to the escalation in the protein content of sprouted seeds [45].

The fat content in lima and adzuki beans decreased from 1.47 (RG) to 1.03% (G72 h) and 3.16 (RG) to 1.95% (G72 h), respectively. The fat content decreased in lima and adzuki beans to 29.93 and 38.29%, respectively, after germination; the results obtained in the current study were similar to the findings of Jayalaxmi et al. [43], who found a 17.85% decrease in fat content after germination treatment. Lima beans are low in fat, and it is further decreased due to fat being utilized during metabolic activities in the germinated seeds [36]. The decrease in fat content during the germination process may also be due to the transformation of fatty acids into carbohydrates through the glyoxylate cycle [46]. It was also proposed that fatty acids become oxidized to carbon dioxide and water to generate energy for germination, leading to the synthesis of certain structural components in young seedlings [42].

The ash and fiber content in lima beans increased from 3.78 (RG) to 4.49% (G72 h) and 6.55 (RG) to 7.94% (G72 h), respectively. The ash and fiber content increased by 18.78 and 21.22%, respectively, after germination. Similarly, the value of the ash and fiber contents in adzuki beans increased by 18.54 and 29.01%, respectively. The increase in ash content may be apparent due to the loss of starch in germination. Ejigui et al. [46] reported an 11% increase in ash content during germination in red kidney beans. Devi et al. [47] reported a 30.46% increase in fiber content during germination in cowpea seeds. The crude fiber consisting of hemicelluloses, lignin, and cellulose enhanced significantly during sprouting as the plant cells synthesized various cellular compounds [48].

The carbohydrate content and calorific value in lima beans decreased from 62.03 (RG) to 56.89% (G72 h) and 350.96 (RG) to 335.95% (G72 h), respectively. The carbohydrate content and calorific value in lima beans decreased by 8.28 and 4.27%, respectively, after soaking and germination. Similarly, the carbohydrate and calorific values in adzuki beans decreased by 20.93 and 11.91%, respectively, after soaking and germination treatments. Similar results were found by Kavitha and Parimalavalli [49], who reported a 20.06 and 9.42% decrease in carbohydrate content and calorific value, respectively, in germinated maize flour. Vidal-Valverde et al. [50] reported that during sprouting, carbohydrate content was used as a source of energy for embryonic growth.

The in vitro protein digestibility (IVPD) in lima and adzuki beans increased by 14.75 and 10.98%, respectively. The results were comparable with the findings of Alonso et al. [32], who observed a 14.53% increase in in vitro protein digestibility in *Phaseolus vulgaris.* Another study by Sharma et al. [51] reported an 11.62% increase in the IVPD in pigeon peas. The increase in IVPD in all process treatments might be due to a reduction in anti-nutrients and disintegration in the structure of some native proteins, including the enzyme inhibitors and lectins. It may be the result of increased phytase activity causing the breakdown of phytic acid in the seeds and augmented α-galactosidase activity to reduce oligosaccharides in seeds. Also, the breakdown of proteins into amino acids resulted in increased digestibility of proteins [52].

#### 3.5.2. Anti-Nutritional Components

The phytic and tannin content in lima beans decreased from 20.48 (RG) to 12.68 (G72) and 10.70 (RG) to 4.35 mg/100 g (G72), respectively. The phytic and tannin contents in adzuki beans decreased from 7.12 (RG) to 3.45 (G72) and 6.36 (RG) to 2.47(G72), respectively. The reduction in phytic and tannin contents in lima beans was 38.08 and 59.34%, respectively, and the same in adzuki beans was reported as 51.54 and 61.16%, respectively, after germination for 72 h. Yasmin et al. [53] found reductions of 42.62 and 68.85% in phytic and tannin content in germinated kidney beans, respectively. Patterson et al. [54] also found a 43% decline in the tannin content in chickpeas after 24 h of germination treatment, and Olika et al. [55] found a 57.35% decrease in phytic acid content in germinated chickpeas. This decline in phytic acid contents can be attributed to an increase in phytase activity during sprouting [56]. The decrease in tannin content after sprouting occurs due to the formation of a hydrophobic association of tannins with seed proteins and enzymes and the leaching of tannins into the water due to a concentration gradient [57].

The trypsin inhibitor activity (TIA) in lima and adzuki beans decreased to 84.22 and 71.01%, respectively, after germination treatment. Jayalaxmi et al. [43] observed a 77.57% decrease in TIA in germinated lima beans. Another report by Patterson et al. [54] found a 76% reduction in TIA in white kidney beans after 5 days of germination. The reduction in TIA after the sprouting might be due to the increased proteolytic activity of enzymes, which become activated during germination, and TIA decreases with an increase in trypsin activity [58].

#### 3.5.3. Antioxidant Activity, Flavonoids, Phenols, Reducing Capacity, and Metal Chelating Activity

There was a significant increase in polyphenols during germination, which contributed significantly to increasing antioxidant activities. The antioxidant activity in lima beans increased from 35.48 (RG) to 47.36% (G72) and 9.74 (RG) to 16.67% (G72) in adzuki beans. The total increase in antioxidant activity in lima and adzuki beans was 33.48 and 71.14%, respectively. Yu-Wei and Wang [37] found an increase of 62.29% in antioxidant activity in adzuki beans. The antioxidant components increased due to the amount of phenolic content that increased in sprouted legumes due to the presence of various hydroxyl groups that behaved like free radical scavengers and resulted in an increase in the antioxidant activity of germinated grains; some bioactive compounds were also found to become increased, and these bioactive compounds acted as an additional antioxidant compound for increasing the antioxidant activity in sprouted beans [59].

The flavonoid content in lima and adzuki beans increased from 20.16 (RG) to 33.63 mg QE/100 g (G72) and 25.38 (RG) to 34.47 mgQE/100 g (G72), respectively. There were 66.81 and 35.81% increases in flavonoid content in lima and adzuki beans, respectively, during 72 h of germination. The results were comparable with the findings of Sharma et al. [46], who reported a 60.14% increase in flavonoid content in germinated pigeon peas. Kaur et al. [60] also found a 21.85% increase in flavonoid content in rice beans during germination.

The reducing capacity in lima and adzuki beans increased from 30.55 (RG) to 45.68% (G72) and 28.77 (RG) to 39.25% (G72), respectively. There were 49.52 and 36.42% increases in the reducing capacity of lima and adzuki beans, respectively, during 72 h of germination. Similar results were found by James et al. [61], who reported a 57.14% increase in reducing capacity in germinated red beans. Khang et al. [39] also found a 42.51% rise in reducing capacity after 5 days of germination. Similarly, the metal chelating activity in lima and adzuki beans increased by 56.06 and 68.23%, respectively. Sharma et al. [51] reported a 62.97% increase in metal chelating activity in germinated pigeon peas. Phytic acid, as phytate, forms strong complexes with many metal ions, thus competing in complexation with EDTA. An increase in the metal chelating activity can be attributed to the increase in the availability of metal ions due to a reduction in phytic acid mass fraction [62].

The phenolic content in lima and adzuki beans increased from 20.48 (RG) to 12.68 mg/g (G72) and 16.16 (RG) to 22.39% (G72), respectively. There were 7.13 and 38.55% increases in the phenolic content of lima and adzuki beans, respectively, during 72 h of germination. Sharma et al. [45] found a 4.7% increase in phenolic content in germinated pigeon peas. Khang et al. [39] also observed a 30.13% increase in phenolic content in adzuki beans. This could be due to the fact that the germinated seeds improved their defensive response by biosynthesis of phenolic compounds, resulting in their survival during germination [63]. Protease also activates and hydrolyzes the protein in the seed during germination. Also, some protein-bound phenolic components were solubilized.

#### 3.5.4. Mineral Contents

The zinc content in lima and adzuki beans increased from 2.45 (RG) to 2.76 ppm (G72) and 4.49 (RG) to 6.74 ppm (G72), respectively, representing 11.83 and 50.11% increase in Zn content of lima and adzuki beans during 72 h of germination, respectively. Similar results were found by Farinde et al. [36] and Kajla et al. [64], who reported a 13.77% increase in zinc content in germinated lima beans and a 50.53% increase in zinc content in germinated flaxseed.

The copper content in lima and adzuki beans increased from 4.17 (RG) to 5.27 ppm (G72) and 1.22 (RG) to 1.51 ppm (G72), respectively, with an increase of 26.37 and 23.77% in Cu content of lima and adzuki beans during 72 h of germination. Similarly, the manganese content increased from 2.36 (RG) to 3.55 ppm (G72) and 1.57 (RG) to 2.22 ppm (G72), respectively. There was an increase of 50.42 and 41.40% in Mn in lima and adzuki beans during 72 h of germination, respectively.

The iron content of the lima and adzuki beans enhanced from 4.04 (RG) to 4.96 ppm (G72) and 5.16 (RG) to 7.32 ppm (G72), respectively, during 72 h of germination. This represents a 23.01 and 41.86% increase in Fe content, respectively. Kajla et al. [64] reported a 15.44% increase in copper content, 37.27 in manganese, and a 37.22% increase in zinc content in germinated flaxseeds. The increase in mineral content during germination and soaking may be due to partial elimination or destruction or both of the anti-nutritional factors, thereby releasing the minerals from their organically bound complexes in the dry seeds [65].

#### 3.5.5. Fourier-Transform Infrared (FTIR) Spectroscopic Analysis

**Figure 3:**
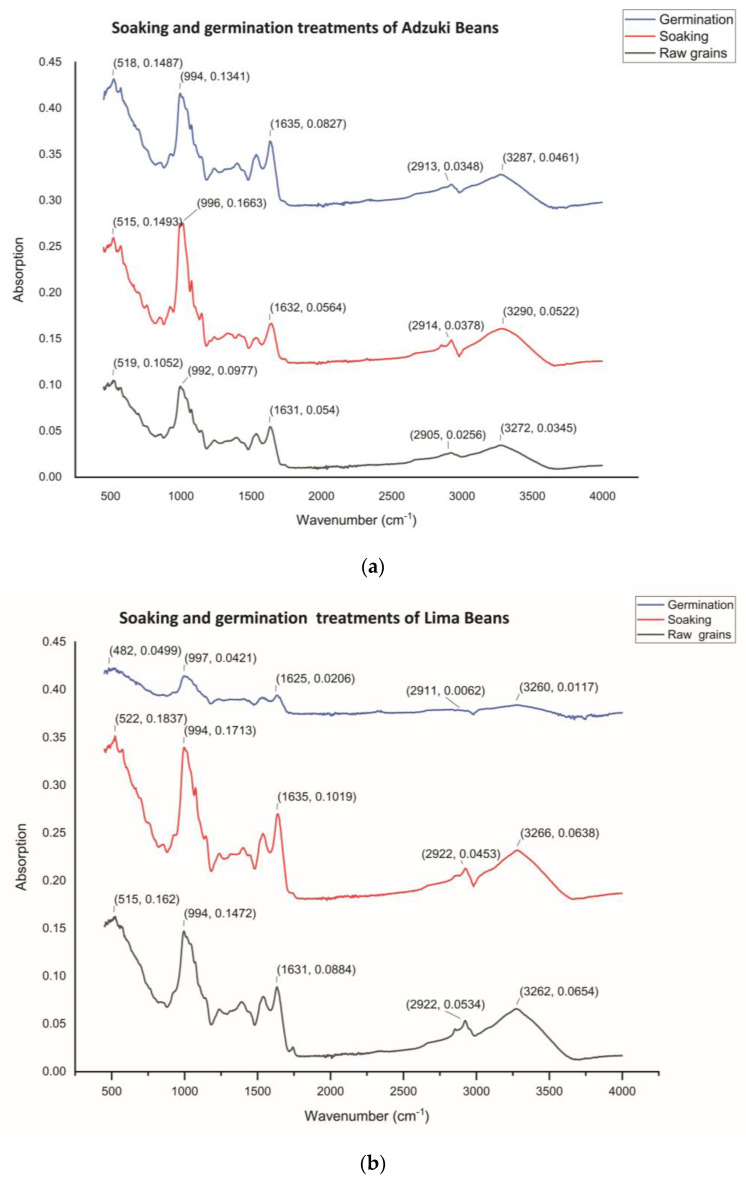
FTIR spectrum of adzuki (**a**) and lima beans (**b**) treated with soaking and germination treatments.

The FTIR spectra of soaking and germination treatments of adzuki beans are given in Figure 3 and Table 5.

The appearance of OD band is shown in the region of 3400–3300, 3300–2500, 1550–1500, 980–960, and 600–500 cm^−1^ in the three FTIR spectra, which shows that these have N-H, O-H, C=O, N-O, C=C, and C-I functional groups, respectively. The N–H stretching with wave no. of 3262, 3266, and 3260 cm^−1^ was reported in raw grain, soaked, and germinated grains, respectively.

Additionally, a peak in absorbance was formed in the wave range of 2500–3300, 1550–1500, 980–960, and 600–500 cm^-1^ as a result of the characteristic O-H stretching, N-O stretching, C=C bending, and C-I stretching, respectively. From the present study, it was observed that soaked grains had higher N-H, O-H, N-O, and C-I stretching compared to C=C bending, which was observed highest in germinated seeds of lima bean grains. It was also observed that raw, germinated, and soaked seeds possessed similar chemical structures. In the carboxylic region, absorption leads to the stretching of the O=H bond, whereas in the aliphatic primary amine region, absorption leads primarily to the stretching of the N–H bond.

## 4. Conclusions and Future Aspects

The current study revealed the impact of processing treatments on the nutritional, anti-nutritional, and bioactive potential of adzuki and lima beans and concluded that there was a significant increase in proteins, minerals, phenolic compounds, in vitro protein digestibility, metal chelating activity, and reducing capacity. Processing treatments like soaking and germination do not require complicated types of equipment, rendering them simple techniques to improve the nutritional and sensory characteristics of germinated grains. Soaking could be one of the processes to remove soluble anti-nutritional factors, which can be eliminated with the discarded soaking solution. During germination, the anti-nutritional factors such as tannins, trypsin inhibitors, and phytic acids decreased with an increase in germination time. Leaching of tannins during soaking and their further degradation during germination and increase in the activity of enzyme phytase resulted in the reduction in anti-nutritional components, thereby increasing mineral availability.

The flour obtained from the germinated beans can be incorporated into various functional bakery products, breakfast bars, snacks, and prebiotic non-dairy beverages, which can be used to eradicate protein deficiency among different sections of society. Due to their high nutritional and bioactive components, these have great potential for improving food and nutritional security and a sustainable and inexpensive meat alternative, especially for those from lower socioeconomic backgrounds and the world’s predominately vegetarian and vegan communities. This can also encourage the scientific community, industry, and government to invest in research and development to increase germinated pulse-based foods. These processing methods can also be used to improve the nutritional and bioactive potential of other pulses while lowering the levels of anti-nutrients. This study will help promote soaking and germination processing as effective treatments for enhancing nutritional quality and reducing anti-nutrients, as well as the increased utilization of these underutilized pulses.

## Figures and Tables

**Table 1:** Physical and functional characteristics of lima and adzuki beans.

Parameters	Lima Beans	Adzuki Beans
Moisture (%)	7.29 ± 0.07 ^b^	12.32 ± 0.08 ^a^
Length (mm)	13.26 ± 0.16 ^a^	4.32 ± 0.03 ^b^
Width (mm)	9.87 ± 0.12 ^a^	2.73 ± 0.32 ^b^
Thickness (mm)	9.16 ± 0.06 ^a^	2.36 ± 0.06 ^b^
1000 Grain Wt. (g)	699.65 ± 0.64 ^a^	61.27 ± 1.05 ^b^
Bulk density (g/cm^3^)	0.93 ± 0.06 ^a^	0.86 ± 0.01 ^a^
Tap density (g/cm^3^)	1.26 ± 0.05 ^a^	1.16 ± 0.03 ^a^
Water Absorption Capacity (mL/g)	2.21 ± 0.05 ^a^	2.89 ± 0.11 ^a^
Oil Absorption Capacity (mL/g)	1.66 ± 0.05 ^a^	1.81 ± 0.11 ^a^
Water Solubility Index (%)	20.23 ± 0.10 ^b^	23.23 ± 0.09 ^a^
Swelling Capacity (%)	82.70 ± 0.46 ^a^	70.06 ± 0.03 ^b^
*L* value	82.34 ± 0.07 ^a^	44.14 ± 0.80 ^b^
a* value	−0.56 ± 0.07 ^b^	4.33 ± 0.10 ^a^
b* value	8.07 ± 0.03 ^a^	0.86 ± 0.04 ^b^

Values are expressed as mean ± SD. Duncan’s LSD post hoc analysis at *p* < 0.05 indicates that values within rows sharing the same letter are not significantly different.

**Table 2:** Principal component analysis and loading of first four components.

Factor Number	Lima Beans				Adzuki Beans			
	1	2	3	4	1	2	3	4
Initial eigenvalues	11.245	6.06	0.000	0.000	11.344	4.66	0.000	0.000
% of variance	0.937	0.050	0.006	0.000	0.945	0.039	0.000	0.000
Cumulative %	93.70	98.80	100	100	94.50	98.40	100	100
**Factor Loadings**								
Moisture content (%)	−0.291	0.166	−0.510	0.503	0.288	0.310	0.297	0.097
Fat content (%)	0.275	**0.452**	−0.576	−0.191	−0.291	0.053	−0.384	**0.632**
Fiber content (%)	−0.282	−0.395	−0.089	0.416	**0.294**	0.078	−0.301	−0.034
Ash content (%)	−0.296	−0.130	0.029	−0.274	0.253	−0.753	0.247	0.069
Protein content (%)	−0.283	0.356	**0.446**	0.290	0.286	**0.363**	−0.225	−0.095
Carbohydrate (%)	0.287	−0.341	0.058	0.294	0.289	−0.241	−0.283	0.420
Calorific value (Kcal/100 g)	**0.296**	0.081	0.043	**0.423**	−0.296	0.066	0.000	0.086
In vitro protein digestibility (%)	−0.292	0.227	0.244	−0.148	0.295	0.088	0.220	−0.100
Phytic acid (mg/100 g)	0.291	−0.274	0.097	−0.057	−0.292	−0.150	−0.302	−0.574
Tannin contents (mg/100 g)	0.278	0.446	0.342	0.204	−0.293	−0.061	**0.451**	0.157
Trypsin-inhibitor activity (TIU/100 g)	0.296	−0.136	0.086	−0.106	−0.291	0.238	0.332	0.138
Antioxidant activity (% DPPH scavenging capacity)	−0.296	−0.037	−0.044	−0.188	0.293	0.207	0.170	0.095

1—PC1; 2—PC2; 3—PC3; 4—PC4; PC—principal component. The values in bold indicates the dominating factor in each principal component.

**Table 3:** Changes in nutritional characteristics of lima beans during soaking and germination treatments.

Parameters	Time (h)					
		Soaking		Germination		
	0	12	24	24	48	72
Moisture content (%)	7.29 ± 0.07 ^d^	7.31 ± 0.01 ^d^	7.38 ± 0.01 ^c^	7.43 ± 0.01 ^c^	7.57 ± 0.02 ^b^	7.74 ± 0.05 ^a^
Protein content (%)	18.59 ± 0.61 ^e^	18.24 ± 0.01 ^e^	19.15 ± 0.01 ^d^	20.07 ± 0.05 ^c^	20.94 ± 0.04 ^b^	21.92 ± 0.04 ^a^
Fat content (%)	1.47 ± 0.25 ^a^	1.36 ± 0.30 ^a^	1.28 ± 0.11 ^ab^	1.23 ± 0.08 ^ab^	1.19 ± 0.08 ^ab^	1.03 ± 0.01 ^b^
Ash content (%)	3.78 ± 0.06 ^b^	4.26 ± 0.63 ^a b^	4.29 ± 0.08 ^ab^	4.34 ± 0.28 ^a^	4.41 ± 0.06 ^a^	4.49 ± 0.06 ^a^
Fiber content (%)	6.55 ± 0.03 ^e^	6.79 ± 0.04 ^d^	6.88 ± 0.06 ^d^	7.37 ± 0.15 ^c^	7.66 ± 0.14 ^b^	7.94 ± 0.05 ^a^
Carbohydrate content (%)	62.03 ± 0.46 ^a^	62.04 ± 0.61 ^b^	61.02 ± 0.11 ^c^	59.56 ± 0.43 ^d^	58.21 ± 0.26 ^e^	56.89 ± 0.05 ^f^
Calorific value (kcal/100 g)	350.96 ± 1.48 ^a^	345.86 ± 3.90 ^b^	344.50 ± 0.72 ^bc^	341.67 ± 1.12 ^cd^	339.14 ± 0.46 ^d^	335.95 ± 0.13 ^e^
Phytic acid (mg/100 g)	20.48 ± 0.13 ^a^	19.20 ± 0.06 ^b^	18.07 ± 0.02 ^c^	17.67 ± 0.42 ^c^	14.58 ± 0.41 ^d^	12.68 ± 0.42 ^e^
Tannin content (mg/100 g)	10.70 ± 0.08 ^a^	9.85 ± 0.05 ^b^	9.05 ± 0.04 ^c^	6.57 ± 0.12 ^d^	5.49 ± 0.06 ^e^	4.35 ± 0.12 ^f^
Phenolic content (mgGAE/100)	110.43 ± 0.16 ^f^	115.27 ± 0.04 ^e^	120.59 ± 0.04 ^d^	125.41 ± 0.07 ^c^	130.32 ± 1.17 ^b^	139.74 ± 0.12 ^a^
Antioxidant activity (% DPPH scavenging capacity)	35.48 ± 0.16 ^f^	36.70 ± 0.08 ^e^	38.59 ± 0.07 ^d^	40.28 ± 0.06 ^c^	43.71 ± 0.02 ^b^	47.36 ± 0.08 ^a^
Trypsin inhibitor (TIU/100 g)	20.41 ± 0.36 ^f^	14.40 ± 0.34 ^e^	13.13 ± 0.12 ^d^	7.68 ± 0.27 ^c^	5.53 ± 0.01 ^b^	3.22 ± 0.09 ^a^
Flavonoid content (mgQE/100 g)	20.16 ± 0.05 ^f^	21.49 ± 0.07 ^e^	23.19 ± 0.10 ^d^	28.63 ± 0.26 ^c^	30.62 ± 0.30 ^b^	33.63 ± 0.14 ^a^
Reducing capacity (%)	30.55 ± 0.05 ^f^	33.66 ± 0.09 ^e^	35.19 ± 0.16 ^d^	39.74 ± 0.16 ^c^	42.21 ± 0.06 ^b^	45.68 ± 0.12 ^a^
Metal chelating activity (%)	35.51 ± 0.07 ^f^	39.06 ± 0.71 ^e^	42.88 ± 0.11 ^d^	47.48 ± 0.16 ^c^	51.80 ± 0.17 ^b^	55.42 ± 0.07 ^a^
In vitro protein digestibility (%)	67.85 ± 0.09 ^f^	69.84 ± 0.09 ^e^	70.80 ± 0.25 ^d^	72.42 ± 0.09 ^c^	74.65 ± 0.08 ^b^	77.86 ± 0.05 ^a^
Zinc (ppm)	2.45 ± 0.03 ^bcd^	2.33 ± 0.02 ^cd^	2.25 ± 0.04 ^d^	2.57 ± 0.04 ^abc^	2.66 ± 0.03 ^ab^	2.76 ± 0.32 ^a^
Iron (ppm)	4.04 ± 0.04 ^e^	4.15 ± 0.03 ^d^	4.25 ± 0.03 ^c^	4.46 ± 0.09 ^b^	4.52 ± 0.08 ^b^	4.96 ± 0.03 ^a^
Copper (ppm)	4.17 ± 0.02 ^d^	4.05 ± 0.03 ^e^	3.97 ± 0.03 ^f^	4.25 ± 0.04 ^c^	4.94 ± 0.03 ^b^	5.27 ± 0.02 ^a^
Manganese (ppm)	2.36 ± 0.03 ^f^	2.16 ± 0.03 ^e^	2.27 ± 0.02 ^d^	3.17 ± 0.02 ^c^	3.34 ± 0.03 ^b^	3.55 ± 0.04 ^a^

The values in the table are displayed as mean ± standard deviation; Duncan’s LSD post hoc analysis at *p* < 0.05 indicates that values within rows sharing the same letter are not significantly different.

**Table 4:** Changes in nutritional characteristics of adzuki beans during soaking and germination treatments.

Parameters	Time (h)					
		Soaking		Germination		
	0	12	24	24	48	72
Moisture content (%)	12.32 ± 0.08 ^f^	12.87 ± 0.02 ^e^	13.43 ± 0.06 ^d^	13.59 ± 0.12 ^c^	13.92 ± 0.06 ^b^	14.57 ± 0.03 ^a^
Protein content (%)	19.56 ± 0.03 ^f^	20.96 ± 0.04 ^e^	21.26 ± 0.03 ^d^	22.76 ± 0.03 ^c^	22.97 ± 0.03 ^b^	23.46 ± 0.02 ^a^
Fat content (%)	3.16 ± 0.02 ^c^	3.26 ± 0.03 ^b^	3.35 ± 0.04 ^a^	2.85 ± 0.03 ^d^	2.54 ± 0.02 ^e^	1.95 ± 0.03 ^f^
Ash content (%)	3.29 ± 0.07 ^e^	3.34 ± 0.04 ^e^	3.41 ± 0.04 ^d^	3.54 ± 0.03 ^c^	3.75 ± 0.02 ^b^	3.90 ± 0.06 ^a^
Fiber content (%)	5.79 ± 0.09 ^d^	5.80 ± 0.06 ^d^	5.91 ± 0.08 ^d^	6.19 ± 0.08 ^c^	6.59 ± 0.08 ^b^	7.47 ± 0.06 ^a^
Carbohydrate content (%)	55.87 ± 0.17 ^a^	53.77 ± 0.06 ^b^	52.48 ± 0.08 ^c^	51.24 ± 0.07 ^d^	49.57 ± 0.09 ^e^	49.32 ± 0.06 ^f^
Calorific value (kcal/100 g)	341.69 ± 0.75 ^a^	339.38 ± 0.33 ^b^	335.94 ± 0.71 ^c^	332.14 ± 0.45 ^d^	323.22 ± 0.21 ^e^	318.42 ± 0.35 ^f^
In vitro protein digestibility	70.56 ± 0.23 ^f^	72.16 ± 0.06 ^e^	73.62 ± 0.16 ^d^	75.89 ± 0.07 ^c^	77.54 ± 0.05 ^b^	78.31 ± 0.06 ^a^
Phytic acid (mg/100 g)	7.12 ± 0.03 ^a^	6.15 ± 0.04 ^b^	5.34 ± 0.02 ^c^	4.66 ± 0.03 ^d^	3.98 ± 0.01 ^e^	3.45 ± 0.03 ^f^
Tannin content (mg/100 g)	6.36 ± 0.01 ^a^	6.22 ± 0.01 ^a^	6.09 ± 0.09 ^a^	5.91 ± 0.04 ^a^	4.20 ± 0.25 ^b^	2.47 ± 0.53 ^c^
Trypsin inhibitor (TIU/100 g)	35.23 ± 0.11 ^a^	30.57 ± 0.04 ^b^	25.21 ± 0.06 ^c^	20.64 ± 0.10 ^d^	15.54 ± 0.09 ^e^	10.21 ± 0.06 ^f^
Phenolic content (mg/g)	16.16 ± 1.30 ^e^	18.06 ± 0.72 ^d^	19.21 ± 0.59 ^c d^	20.17 ± 0.33 ^bc^	21.27 ± 1.28 ^a b^	22.39 ± 0.72 ^a^
Antioxidant Activity (% DPPH scavenging capacity)	9.74 ± 0.04 ^c d^	9.92 ± 0.06 ^c d^	10.04 ± 0.03 ^cd^	12.24 ± 0.03 ^bc^	14.98 ± 0.02 ^ab^	16.67 ± 0.02 ^a^
Reducing capacity (%)	28.77 ± 0.02 ^f^	30.04 ± 0.03 ^e^	32.95 ± 0.03 ^d^	35.27 ± 0.02 ^c^	37.65 ± 0.04 ^b^	39.25 ± 0.02 ^a^
Flavonoid content (mg QE/100 g)	25.38 ± 0.18 ^f^	26.89 ± 0.05 ^e^	28.29 ± 0.07 ^d^	30.64 ± 0.17 ^c^	32.34 ± 0.10 ^b^	34.47 ± 0.31 ^a^
Metal chelating activity (%)	20.59 ± 0.05 ^f^	23.80 ± 0.14 ^e^	26.60 ± 0.33 ^d^	28.62 ± 0.12 ^c^	31.52 ± 0.43 ^b^	34.64 ± 0.11 ^a^
Zinc (ppm)	4.49 ± 0.04 ^f^	4.92 ± 0.05 ^e^	5.23 ± 0.04 ^d^	5.94 ± 0.05 ^c^	6.39 ± 0.05 ^b^	6.74 ± 0.04 ^a^
Iron (ppm)	1.57 ± 0.02 ^e^	1.61 ± 0.02 ^e^	1.73 ± 0.05 ^d^	1.85 ± 0.03 ^c^	1.97 ± 0.03 ^b^	2.22 ± 0.03 ^a^
Copper (ppm)	1.22 ± 0.02 ^a^	1.49 ± 0.35 ^a^	1.35 ± 0.07 ^a^	1.41 ± 0.11 ^a^	1.45 ± 0.04 ^a^	1.51 ± 0.02 ^a^
Manganese (ppm)	5.16 ± 0.04 ^f^	5.35 ± 0.04 ^e^	5.51 ± 0.06 ^d^	6.34 ± 0.11 ^c^	6.82 ± 0.07 ^b^	7.32 ± 0.03 ^a^

The values in the table are displayed as mean ± standard deviation; Duncan’s LSD post hoc analysis at *p* < 0.05 indicates that values within rows sharing the same letter are not significantly different.

**Table 5:** Band assignment of raw, soaked, and germinated grains of adzuki and lima beans.

Wave Range (cm^−1^)	Functional Group	Compound Class	Adzuki Beans			Lima Beans		
			Raw Grains	Soaking	Germination	Raw Grains	Soaking	Germination
3400–3300	N-H stretching	Aliphatic primary amine	3272	3290	3287	3260	3266	3262
3300–2500	O-H stretching	Carboxylic acid	2905	2914	2913	2911	2922	2922
1550–1500	N-O stretching	Nitro compound	1631	1632	1635	1625	1635	1631
980–960	C=C bending	Alkene	992	996	994	997	994	994
600–500	C-I stretching	Halo compound	519	515	518	482	522	515

## Data Availability

The original contributions presented in the study are included in the article, further inquiries can be directed to the corresponding authors.
